# Measuring changes in prevalence of hypertension and diabetes across 720 districts in India using cross-sectional data from 2016 to 2021

**DOI:** 10.1136/bmjph-2025-002653

**Published:** 2025-10-10

**Authors:** Anoop Jain, Mayanka Ambade, Shalem Balla, Avnish Pal, Sunil Rajpal, Rockli Kim, S V Subramanian

**Affiliations:** 1 Environmental Health, Boston University, Boston, Massachusetts, USA; 2 Indian Institute of Technology Mandi, Mandi, Himachal Pradesh, India; 3 Department of Economics, FLAME University, Pune, Maharashtra, India; 4 Centre for Research in Wellbeing and Happiness, FLAME University, Pune, Maharashtra, India; 5 Division of Health Policy and Management, Korea University, Seongbuk-gu, Korea (the Republic of); 6 Interdisciplinary Program in Precision Public Health, Department of Public Health Sciences, Graduate School of Korea University, Seoul, Korea (the Republic of); 7 Harvard Center for Population and Development Studies, Cambridge, Massachusetts, USA; 8 Department of Social and Behavioral Sciences, Harvard T H Chan School of Public Health, Boston, Massachusetts, USA

**Keywords:** Community Health, Cardiovascular Diseases, Public Health

## Abstract

**Introduction:**

The age of onset for hypertension and diabetes has been declining in India. However, the extent to which the burden of these two non-communicable illnesses among younger adults varies geographically within India is not known. Thus, the purpose of this study is to examine changes in prevalence of hypertension and diabetes among women and men between the ages of 15 and 49 across India’s 720 districts from 2016 to 2021.

**Methods:**

The study uses repeated cross-sectional data from 2016 and 2021, representative at the district level in India. We analysed women and men between the ages of 15 and 49 for hypertension and diabetes. We used WHO cut-offs for hypertension (systolic blood pressure of 140 mmHg or greater, and/or diastolic blood pressure of 90 mmHg or higher) and diabetes (random blood glucose level of 200 mg/dL or higher). We estimated four-level logistic regression models to derive the district-level prevalence estimates for 720 districts for each outcome for women and men allowing us to assess geographical outcome patterns.

**Results:**

We found considerable variation in rates of hypertension and diabetes across India’s 720 districts among women and men between the ages of 15 and 49. The prevalence of hypertension and diabetes increased in hundreds of districts between 2016 and 2021. We also found a moderately negative relationships between the district-level prevalence of hypertension in 2016 and the change in district-level hypertension between 2016 and 2021 among both women and men. Similarly, there was a slight positive relationship between the district-level prevalence of diabetes among men in 2016 and the district-level change in diabetes between 2016 and 2021. Finally, we did not find strong evidence of a relationship between the district-level prevalence of hypertension and diabetes in either 2016 or 2021. This was the case for both women and men.

**Conclusion:**

Our results highlight the need for regional investments in hypertension and diabetes detection, awareness, treatment and prevention programmes that are tailored to the various risk factors in these different regions.

**WHAT IS ALREADY KNOWN ON THIS TOPIC**

- The prevalence of hypertension and diabetes in India is rising, with a concerning decline in the age of onset. However, district-level data on changes in their prevalence, especially in reproductive-aged populations, remains scarce.

**WHAT THIS STUDY ADDS**

- This study provides granular, district-level insights into the prevalence of hypertension and diabetes from 2016 to 2021 in 720 districts of India. It highlights significant geographical variations, with worsening trends in certain coastal and eastern regions, while others show improvement.

**HOW THIS STUDY MIGHT AFFECT RESEARCH, PRACTICE OR POLICY**

- The findings emphasise the need for localised, gender-specific interventions and monitoring systems. This could inform public health strategies and policy decisions to address non-communicable diseases in India more effectively.

## Introduction

 Hypertension and diabetes are major risk factors and causes for global mortality.^1 2^ These conditions are linked to numerous long-term complications that include reproductive and pregnancy-related issues, premature mortality, disability, accelerated ageing and greater vulnerability to comorbidities.^3^,^9^ Further, the early onset of these conditions, specifically in reproductive ages, adversely impacts educational and economic outcomes, reducing earning potential and imposing prolonged and significant healthcare costs due to the extended duration of the disease thus jeopardising multiple Sustainable Development Goals.^10 11^

The burden of hypertension and diabetes has increased throughout India over the past several decades. For example, the disability-adjusted life years attributable to hypertension increased from 21 million in 1990 to 39 million in 2016.^12^ Similarly, between 1990 and 2016, the number of people with diabetes in India increased from 26 million to 65 million.^13^ Furthermore, the prevalence of these outcomes varies geographically within India.

However, district-level changes in these two outcomes are less understood. Filling this gap is important as prior studies have shown considerable geographical variation in these outcomes. Using data from the National Family Health Survey (NFHS) in 2021, Varghese *et al* show substantial variation in hypertension across and within India’s 36 states and Union Territories.^14^ The prevalence of diabetes also varies across India’s 36 states and Union Territories.^13^ These variations suggest that changes in rates of these two non-communicable diseases (NCDs) might not be uniform across India.

Furthermore, less is known about how these rates have changed subnationally among younger adults still in their reproductive years. Filling this gap is important as the global nutrition transition, especially in India, has led to increased reliance on carbohydrate-rich diets and processed foods, which are strongly linked to elevated blood sugar and pressure.^15^,^18^ These dietary habits are particularly prevalent among younger populations.^19 20^ Additionally, self-care practices such as regular physical activity and consistent healthcare utilisation are often deprioritised in the reproductive age group, exacerbating their vulnerability to hypertension and diabetes.^21^,^23^

Therefore, using cross-sectional data from India’s NFHS in 2016 and 2021, this study examined changes in the prevalence of hypertension and diabetes across 720 districts. We examined these changes for women and men between the ages of 15 and 49. This study is policy relevant given that India’s National Health Mission has emphasised the early identification and sustained treatment of NCDs. Therefore, identifying which districts in India are experiencing increases in hypertension and diabetes prevalence among women and men between 15 and 49 can help policymakers target the appropriate intervention strategies.

## Materials and methods

### Data and sampling strategy

This study analysed data from the fourth and fifth rounds of India’s NFHS, conducted in 2015–2016 (NFHS-4) and 2019–2021 (NFHS-5), respectively.^24 25^ For simplicity, the analysis references only the end year of each survey. Both surveys are part of the Demographic and Health Surveys (DHS) programme and collect extensive data on population health, nutrition and well-being, including indicators related to barriers in accessing healthcare. The survey design employed a two-stage sampling approach. In the first stage, clusters, which were villages in rural communities and Census Enumeration Blocks in urban communities, were selected within each district. The number of clusters was determined using probability proportional to size within each district of a given state/Union Territory. In the second stage, households were randomly selected within each cluster. A complete description of the sampling strategy is available in the latest round report (fifth round) of the NFHS.^26^

### Outcomes

We analysed the prevalence of hypertension and diabetes among women and men between the ages of 15 and 49. Those with a systolic blood pressure of 140 mmHg and above, and/or those with a diastolic blood pressure of 90 mmHg and above were classified as having hypertension. These cut-offs are based on the WHO definition of hypertension and this cut-off does not vary by age or gender.^27^ The systolic and diastolic blood pressure was measured three times for each participant using a portable blood pressure monitor (HEM-8712, Omron Healthcare).^25^ Those with a random blood glucose reading of 200 mg/dL and above were classified as diabetic. Again, this cut-off is based on the WHO definition for random glucose level, which is measured at any time of day regardless of when the individual last ate.^28^ This cut-off does not vary by age or gender.^28^ Blood glucose was measured with a portable blood glucose measuring instrument (FreeStyle Optium H, Abbott Laboratories).^25^

### District geometry

The clusters from NFHS-4 and NFHS-5 were reassigned to align with updated geographical boundaries, ensuring they corresponded accurately to 720 districts. For the majority of districts, the cluster-to-district linkages provided in the DHS microdata were retained unchanged for all NFHS-5 clusters, except for those in Andhra Pradesh. Similarly, for NFHS-4 clusters, no adjustments were made where district boundaries remained consistent between NFHS-4 and NFHS-5. Consequently, the original cluster-to-district linkages were preserved for 694 districts in NFHS-5 and 564 districts in NFHS-4 without requiring modifications. Further details are available in previously published work.^29^

### Statistical analysis

A four-level logistic regression model was used to compute precision-weighted predicted probabilities of hypertension and diabetes at the cluster level. The hierarchical structure includes individuals i (level 1) nested within clusters j (level 2), which are nested within districts k (level 3) and further nested within states l (level 4) as shown in (1):


$$Y_{ijkl}=\;\beta_{0}+\left(u_{jkl}+\;v_{kl}+\;f_{l}\right)$$

In the aforementioned model, $u_{jkl}$, $v_{kl},f_{l}$ are model residuals specific to cluster, district and state, respectively. These residuals are assumed to have a normal distribution with a mean of 0 and a variance as shown in (2):


$$u_{jkl}∼\left(0,\sigma_{u}^{2}\right);v_{kl}∼\left(0,\sigma_{v}^{2}\right);f_{l}∼\left(0,\sigma_{f}^{2}\right)$$

Here the term $\sigma_{u}^{2}$ denotes within-district, inter-cluster variation, $\sigma_{v}^{2}$ denotes within-state, inter-district variation and $\sigma_{f}^{2}$ stands for inter-state variation. Variance across individual men and women is assumed to follow a logistic distribution with a fixed variance of π^2/3^ or 3.29.

Multilevel modelling was performed using the Stata V.18 and MLwiN V.3.0 software programme (using *runmlwin*) and the Monte Carlo Markov Chain (MCMC) method using the Gibbs sampler, keeping the default prior distribution of iterated generalised least square as the starting value.^30^,^32^ We used the MCMC approach as this provides more reliable estimates than other quasi-likelihood approaches.^33 34^ The district-level prevalence (in %) of each indicator is computed by taking the simple average of the cluster-level predicted probabilities for each district. This methodology was applied consistently across both NFHS-4 and NFHS-5 data to estimate the prevalence for indicators for all 720 districts. A full description of the MCMC output is provided in online supplemental tables 1 and 2.

### Ethics statement

This study used publicly accessible secondary data obtained from the DHS website. DHS data are available at https://dhsprogram.com (requiring a simple application). The DHS data are not collected specifically for this study and no one on the study team has access to identifiers linked to the data. These activities do not meet the regulatory definition of human subject research. As such, an Institutional Review Board (IRB) review is not required. The Harvard Longwood Campus IRB allows researchers to self-determine when their research does meet the requirements for IRB oversight via guidance online using an IRB Decision Tool.

### Patient and public involvement statement

It was not appropriate or possible to involve patients or the public in the design, conduct, reporting, or dissemination plans of our research.

### Role of the funding source

This research was funded by the Bill and Melinda Gates Foundation, INV-002992. The funder had no involvement in any facet of this study, including study design, data analysis and interpretation or manuscript writing.

## Results

### Sample characteristics

In 2016, we analysed 661 757 women and 96 337 men for hypertension prevalence, and 684 845 women and 100 019 men for diabetes prevalence. In 2016, the weighted prevalence of hypertension among women was 9.2% (95% CI: 9.1% to 9.2%), and the weighted prevalence of hypertension among men was 13.5% (95% CI: 13.3% to 13.7%). In 2016, the weighted prevalence of diabetes among women was 12.1% (95% CI: 12.0% to 12.2%) and the weighted prevalence of diabetes among men was 15.7% (95% CI: 15.5% to 15.9%). In 2021, we analysed 651 810 women and 81 193 men for hypertension, and 690 748 women and 86 692 men for diabetes. The weighted mean prevalence of hypertension was 9.8% (95% CI: 9.7% to 9.9%) among women and 15.3% (95% CI: 15.0% to 15.5%) among men in 2021. The weighted mean prevalence of diabetes was 17.3% (95% CI: 17.2% to 17.5%) among women and 22.5% (95% CI: 22.2% to 22.7%) among men in 2021. These results are presented in table 1 and are descriptive values from the sample while all values presented hereafter are results from our model estimates. A complete description of the number of districts and individuals per state/Union Territory is presented in online supplemental table 3.

**Table 1:** Distribution of sample and prevalence rates of hypertension and diabetes among reproductive-aged adults (15–49 years) in 2016 and 2021

	Hypertension			Diabetes		
	2016	2021	Change in prevalence	2016	2021	Change in prevalence
	n (prevalence)	n (prevalence)		n (prevalence)	n (prevalence)	
Women	661 757 (9.2)	651 810 (9.8)	0.6	684 845 (12.1)	690 748 (17.3)	5.2
Men	96 337 (13.5)	81 193 (15.3)	1.8	100 019 (15.7)	86 692 (22.5)	6.8

**Figure 1:**
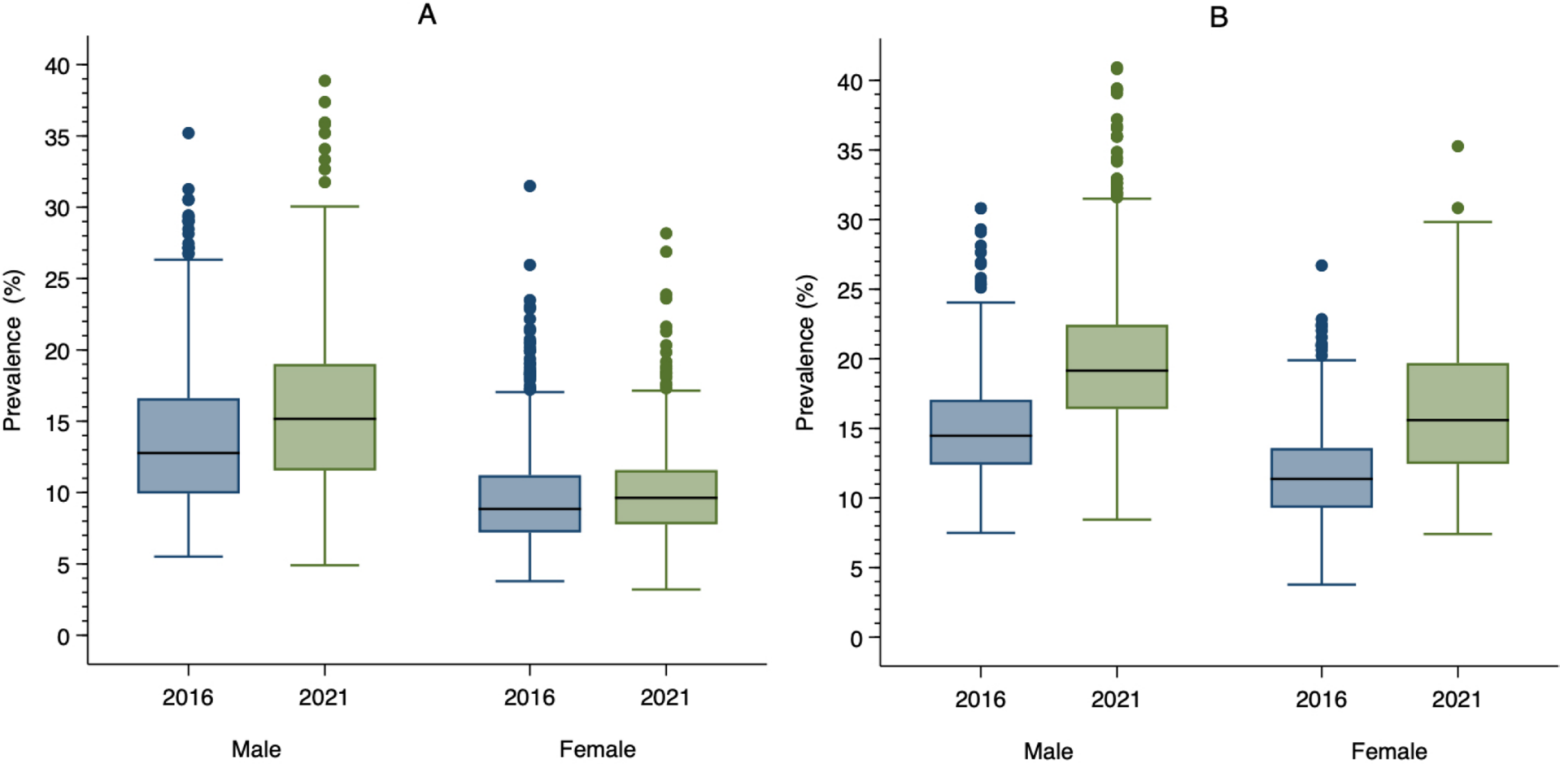
(**A**) Distribution of district-level prevalence of hypertension among reproductive aged (15–49 years) adults in 2016 (navy) and 2021 (forest green). (**B**) Distribution of district-level prevalence of diabetes among reproductive aged (15–49) adults in 2016 (navy) and 2021 (forest green). Note: Hypertension is defined as systolic blood pressure ≥140 mmHg and/or diastolic blood pressure ≥90 mmHg. Diabetes is defined as blood glucose level ≥200 mg/dL. The horizontal bar inside the box indicates the median, the lower and upper ends of the boxes are the 25th and 75th percentile, respectively, and represent the IQR. The whiskers indicate data 1.5 times the IQR and the circles indicate outliers.

The median district-level prevalence of hypertension in women in 2016 was 8.9% and this increased to 9.6% in 2021. The IQR of the district-level prevalence of hypertension in women decreased from 4.0 percentage points to 3.8 percentage points between 2016 and 2021. Among men, the median district-level prevalence of hypertension was 12.8% in 2016 and 15.2% in 2021. The IQR of the district-level prevalence of hypertension in men increased from 6.7 percentage points to 7.5 percentage points between 2016 and 2021. The median district-level prevalence of diabetes was 11.4% in 2016 and 15.6% in 2021 among women. The IQR of the district-level prevalence of diabetes in women increased from 4.3 percentage points to 7.2 percentage points between 2016 and 2021. Among men, the median district-level prevalence of hypertension was 14.5% in 2016 and 19.1% in 2021. The IQR of the district-level prevalence of diabetes among men increased from 4.7 percentage points to 6.0 percentage points between 2016 and 2021. These results are presented in figures1  2, and online supplemental figures 1–4.

**Figure 2:**
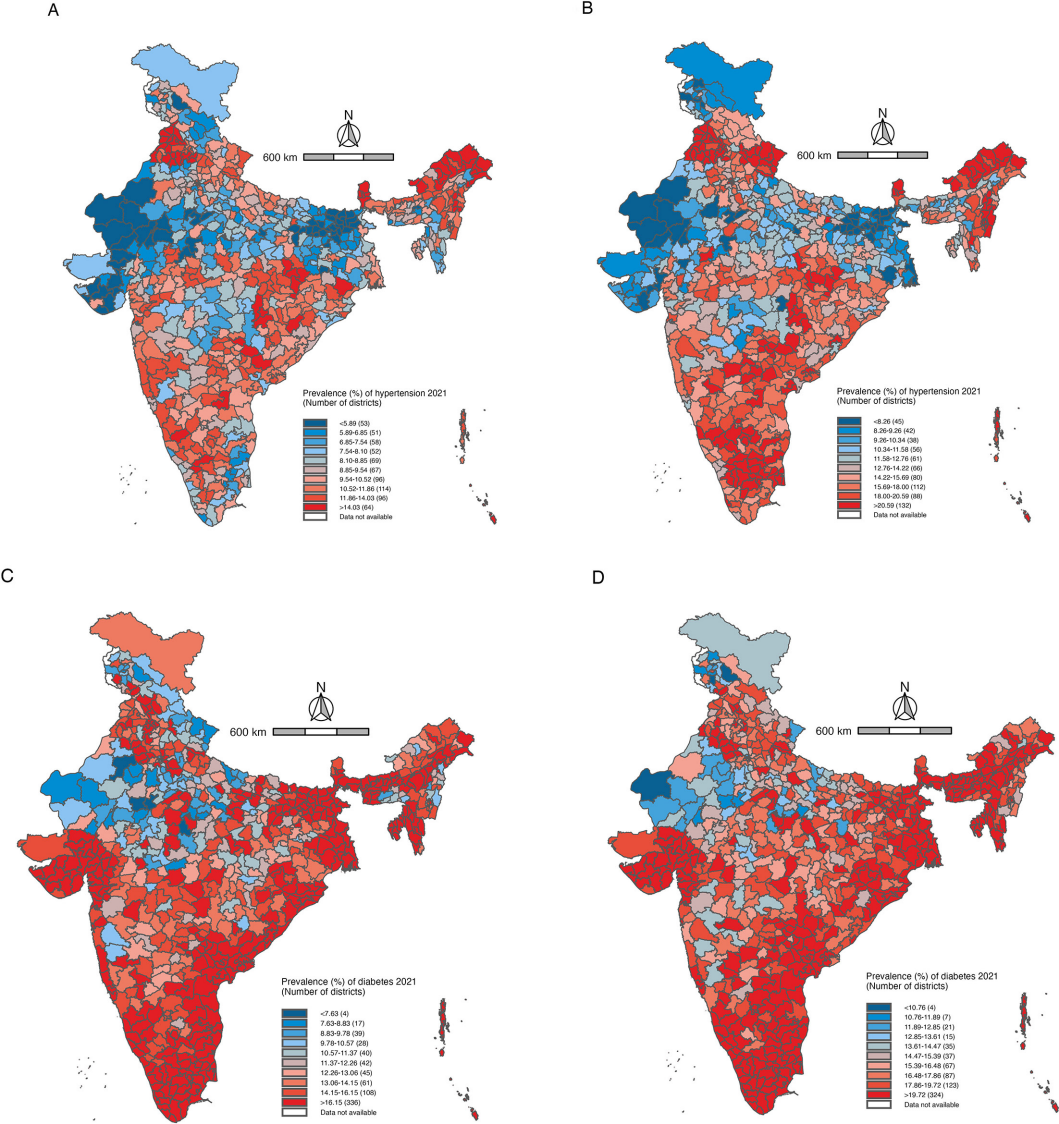
(**A**) District-level prevalence of hypertension among reproductive aged women (15–49 years) in India, 2021. (**B**) District-level prevalence of hypertension among reproductive aged men (15–49 years) in India, 2021. (**C**) District-level prevalence of diabetes among reproductive aged women (15–49 years) in India, 2021. (**D**) District-level prevalence of diabetes among reproductive aged men (15–49 years) in India, 2021.

### Changes in district mean of hypertension and diabetes

**Figure 3:**
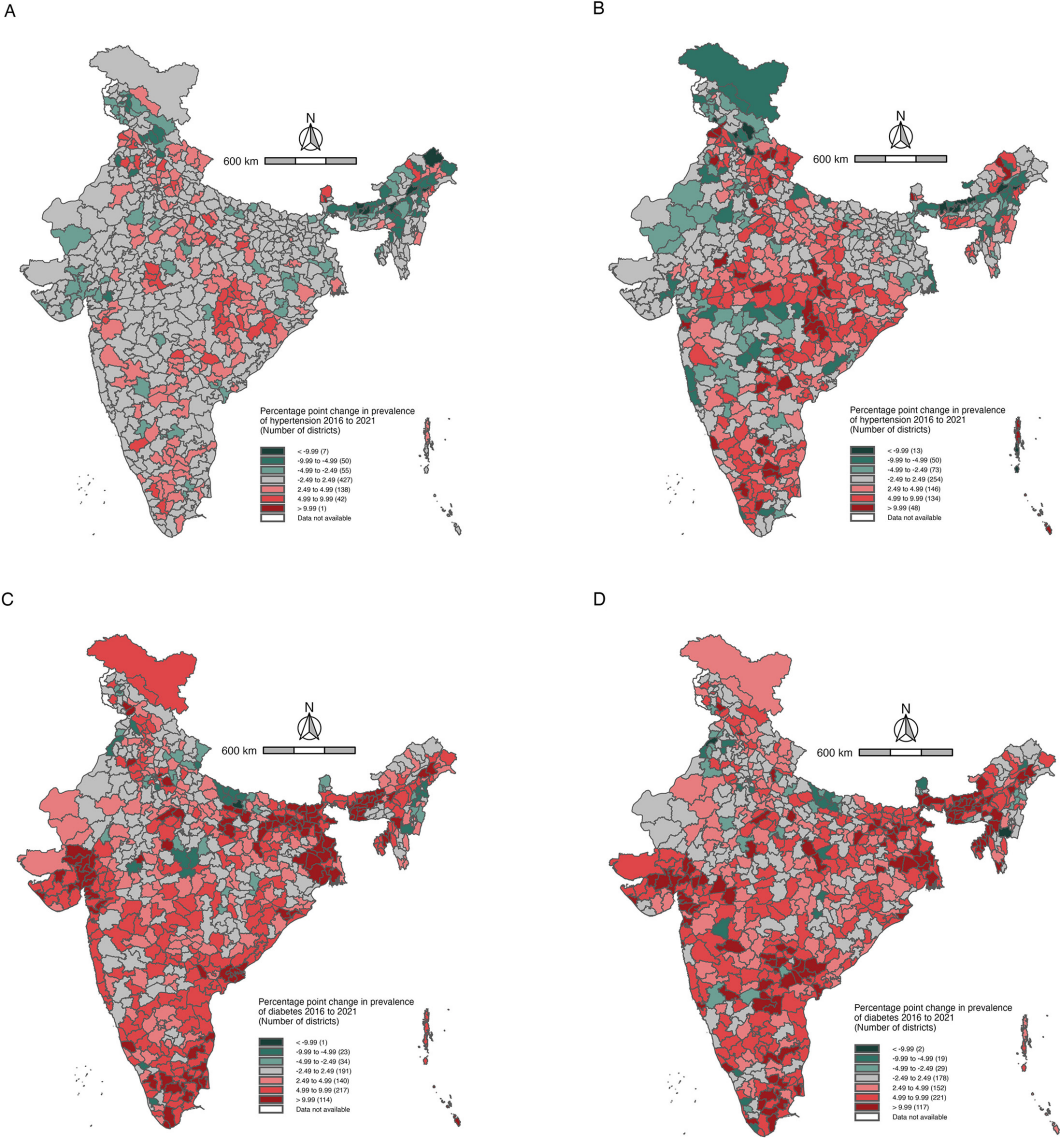
(**A**) District-level percentage point change in prevalence of hypertension among reproductive aged women (15–49 years) 2016–2021 in India. (**B**) District-level percentage point change in prevalence of hypertension among reproductive aged men (15–49 years) 2016–2021 in India. (**C**) District-level percentage point change in prevalence of diabetes among reproductive aged women (15–49 years) 2016–2021 in India. (**D**) District-level percentage point change in prevalence of diabetes among reproductive aged men (15–49 years) 2016–2021 in India.

We found that the prevalence of hypertension for women changed between −2.49 and 2.49 percentage points in 427 districts from 2016 to 2021. The prevalence of hypertension for women increased by more than 2.49 percentage points in 181 districts between 2016 and 2021, and decreased by more than 2.49 percentage points in 112 districts during the same time. Among men, the prevalence of hypertension changed between −2.49 and 2.49 percentage points in 254 districts from 2016 to 2021. The prevalence of hypertension among men increased by more than 2.49 percentage points in 328 districts between 2016 and 2021, and decreased by more than 2.49 percentage points in 136 districts during the same time. These results are presented in figure 3.

We found that the prevalence of diabetes among women changed between −2.49 and 2.49 percentage points in 191 districts between 2016 and 2021. The prevalence of diabetes among women increased by more than 2.49 percentage points in 471 districts between 2016 and 2021, and decreased by more than 2.49 percentage points in 58 districts during the same time. Among men, the prevalence of diabetes changed between −2.49 and 2.49 percentage points in 178 districts between 2016 and 2021. The prevalence of diabetes among men increased by more than 2.49 percentage points in 490 districts between 2016 and 2021, and decreased by more than 2.49 percentage points in 50 districts during that same time. These results are presented in figure 3.

### District correlations between hypertension and diabetes

**Figure 4:**
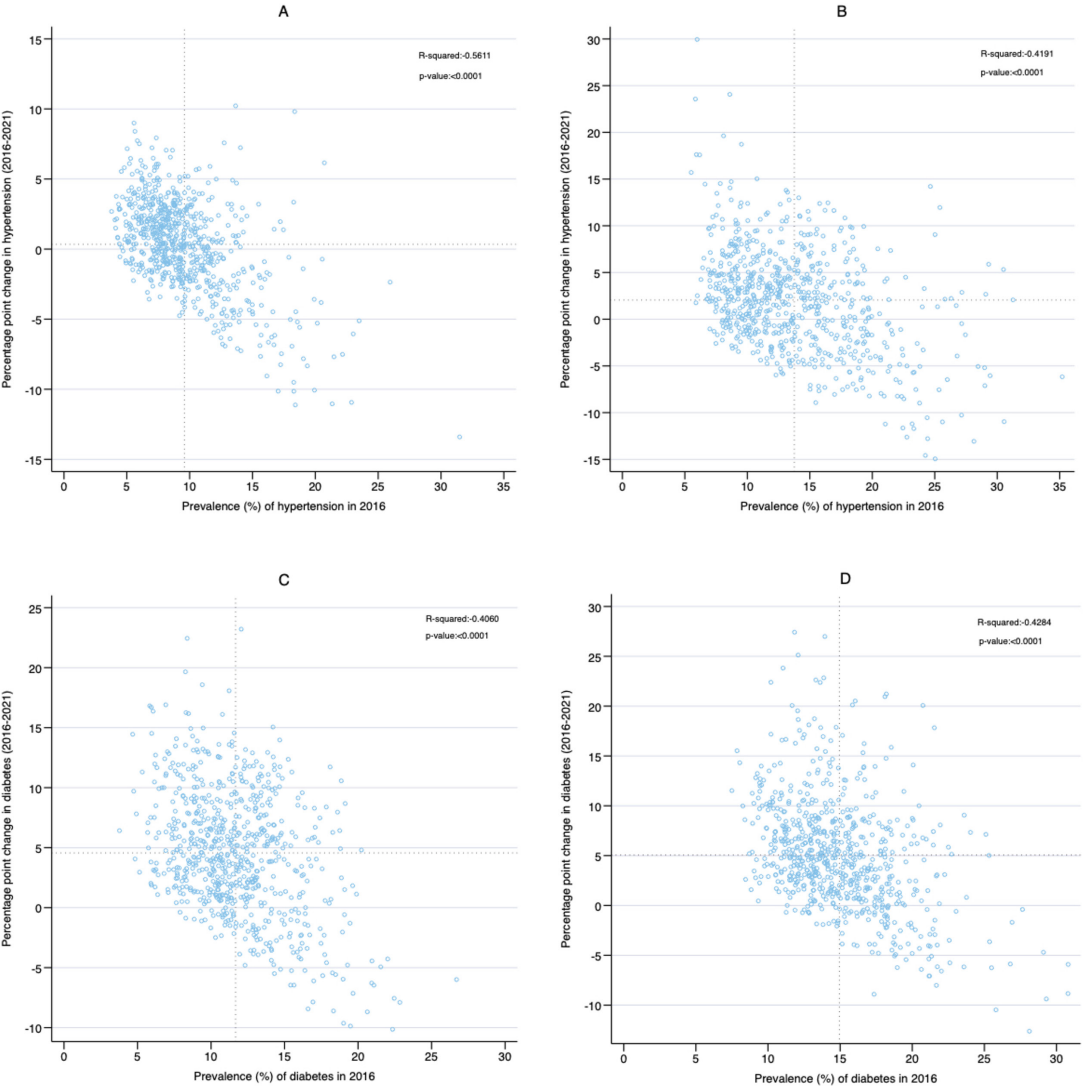
(**A**) District-level association between the prevalence of hypertension in 2016 and the change in hypertension prevalence among reproductive aged women (15–49 years) between 2016 and 2021. (**B**) District-level association between the prevalence of hypertension in 2016 and the change in hypertension prevalence among reproductive aged men (15–49 years) between 2016 and 2021. (**C**) District-level association between the prevalence of diabetes in 2016 and the change in diabetes prevalence among reproductive aged women (15–49 years) between 2016 and 2021. (**D**) District-level association between the prevalence of diabetes in 2016 and the change in diabetes prevalence among reproductive aged men (15–49 years) between 2016 and 2021.

In 2016, the average district-level prevalence of hypertension was 9.5% among women. We found that 293 districts had an above-average district-level prevalence of hypertension. Of these districts, 112 experienced an increase in hypertension prevalence, while 181 districts experienced a decrease in hypertension prevalence between 2016 and 2021. Conversely, 427 districts had a below-average district-level prevalence of hypertension. We found that 307 districts experienced an increase in hypertension prevalence, whereas 120 districts experienced a decrease in hypertension prevalence during the same period. These results are presented in figure 4.

In 2016, the average district-level prevalence of hypertension was 13.8% among men. We found that 305 districts had an above-average district-level prevalence of hypertension. Of these districts, 158 experienced an increase in hypertension prevalence, while 147 districts experienced a decrease in hypertension prevalence between 2016 and 2021. Conversely, 413 districts had a below-average district-level prevalence of hypertension. We found that 309 districts experienced an increase in hypertension prevalence, while 104 districts experienced a decrease in hypertension prevalence during the same period. These results are presented in figure 4.

In 2016, the average district-level prevalence of diabetes was 11.7% for women. We found that 328 districts had an above-average district-level prevalence of diabetes. Of these districts, 230 districts experienced an increase in diabetes prevalence, while 98 districts experienced a decrease in diabetes prevalence between 2016 and 2021. Conversely, 392 districts had a below-average district-level prevalence of diabetes. We found that 364 districts experienced an increase in diabetes prevalence, whereas 28 districts experienced a decrease in diabetes prevalence during the same period. These results are presented in figure 4.

In 2016, the average district-level prevalence of diabetes was 14.9% among men. We found that 321 districts had an above-average district-level prevalence of diabetes. Of these districts, 227 experienced an increase in diabetes prevalence, while 94 districts experienced a decrease in diabetes prevalence between 2016 and 2021. Conversely, 397 districts had below-average district-level prevalence of diabetes. Of these districts, 380 experienced an increase in diabetes prevalence, while 17 districts experienced a decrease in diabetes prevalence during the same period. These results are presented in figure 4.

In 2016 and 2021, we did not find strong correlations between the district-level prevalence of diabetes and hypertension. This was true for both women and men. These results are presented in online supplemental figures 5–8.

## Discussion

Our study had three salient findings. First, we found considerable between-district variations in the prevalence of hypertension and diabetes among women and men between the ages of 15–49 in India. Second, the prevalence of hypertension and diabetes increased in hundreds of districts across India between 2016 and 2021. Third, we found slight negative relationships between the district-level prevalence of hypertension in 2016 and the change in district-level hypertension between 2016 and 2021 among both women and men. This was also true for the prevalence of diabetes for both women and men. Finally, we did not find strong evidence of a relationship between the district-level prevalence of hypertension and diabetes in either 2016 or 2021. This was true for both women and men.

There are four data-related limitations with this study. First, we only used the random glucose cut-off of 200 mg/dL to classify people with or without diabetes. As such, it is possible that our prevalence estimates are conservative as we did not consider fasting glucose measurements or whether or not individuals had been previously told by a doctor they have elevated blood sugar or diabetes. Second, the analysis treats the entire reproductive age range as a homogeneous group. However, susceptibility to risk factors varies considerably within this age range, and an age-stratified analysis could provide more nuanced and accurate estimates. Third, the time period between the two study years spans only 3–5 years. Despite this relatively short interval, it is sufficient for assessing the identification and management of hypertension and diabetes, as effective medical interventions can quickly control these conditions. Fourth, missing values excluded from the analysis may be systematically distributed, as certain segments of the population might be less willing to provide blood samples or undergo biomarker testing. Nevertheless, given that missing values constitute only a small proportion of the sample, their exclusion is unlikely to significantly impact the overall estimates. Lastly, estimates for districts in Andhra Pradesh may be underpowered as the geographical realignment created 26 districts from 13 districts.

Despite these limitations, this study provides critical insights into trends in hypertension and diabetes among women and men across India’s 720 districts. We found that in many districts, the prevalence of hypertension has decreased among women and men. This could suggest improved management of the condition and the fact that blood pressure monitoring has improved in many parts of the country.^35^,^37^ While there remain significant missed opportunities for detecting high blood pressure, especially among older adults, the decline among reproductive-aged individuals may indicate better identification or improved medication adherence.^38^ However, we also found that in many districts, the prevalence of hypertension has increased among both women and men between the ages of 15 and 49. It is possible this is a consequence of the geographical disparities in hypertension awareness, treatment and control that persist throughout India.^39 40^ Programmes such as the India Hypertension Control Initiative, which has been piloted in several districts and states, have been shown to improve detection and control of hypertension.^41^ Programmes such as this should be scaled out nationwide in an effort to detect, prevent and treat hypertension.

Our results also show geographical variation in diabetes prevalence among women and men throughout India. This has been shown in a previous study,^42^ and could be due to variations in access to healthcare facilities and provider quality. These variations could also be due to the fact that risk factors for diabetes, such as obesity, tobacco use and alcohol consumption also vary geographically within India.^43^ We also show that rates of diabetes increased in most districts for women and men between 2016 and 2021. Diabetes detection and awareness remains extremely poor throughout India.^44^ Health system performance in terms of detecting and managing diabetes also varies considerably throughout India, and the system remains the weakest in rural communities.^44^ These factors could explain why the prevalence has increased throughout India.

## Supplementary material

10.1136/bmjph-2025-002653 online supplemental file 1

## Data Availability

Data are available through the DHS programme website (https://dhsprogram.com/)
