# Supplementary material for: Measuring changes in prevalence of hypertension and diabetes across 720 districts in India using cross-sectional data from 2016 to 2021
Source: BMJ Public Health. 2025 Oct 10;3(2):e002653. doi: 10.1136/bmjph-2025-002653 (PMC12542712; doi:10.1136/bmjph-2025-002653)

**Supplementary tables and figures**

| **Table of Contents** | | |
| --- | --- | --- |
| **Table/Figure** | **Title** | **Page** |
| **Table S1** | Markov Chain Monte Carlo (MCMC) model for hypertension across individual, state, district, and cluster among reproductive aged (15-49 years) adults in India in 2016 and 2021. | 2 |
| **Table S2** | Markov Chain Monte Carlo (MCMC) model for diabetes across individual, state, district, and cluster among reproductive aged (15-49 years) adults in India in 2016 and 2021 | 3 |
| **Table S3** | Distribution of sample of hypertension and diabetes among reproductive aged (15-49 years) among states and union territories of India in 2016 and 2021 | 4 |
| **Figure S1** | District-level prevalence of hypertension among reproductive aged women (15-49 years) in India, 2016 | 5 |
| **Figure S2** | District-level prevalence of hypertension among reproductive aged men (15-49 years) in India, 2016 | 6 |
| **Figure S3** | District-level prevalence of diabetes among reproductive aged women (15-49 years) in India, 2016 | 7 |
| **Figure S4** | District-level prevalence of diabetes among reproductive aged men (15-49 years) in India, 2016. | 8 |
| **Figure S5** | District level association between prevalence of hypertension and diabetes among reproductive aged women (15-49) in 2021 | 9 |
| **Figure S6** | District level association between prevalence of hypertension and diabetes among reproductive aged women (15-49) in 2016 | 10 |
| **Figure S7** | District level association between prevalence of hypertension and diabetes among reproductive aged men (15-49) in 2021 | 11 |
| **Figure S8** | District level association between prevalence of hypertension and diabetes among reproductive aged men (15-49) in 2016 | 12 |

**Table S1**: Markov Chain Monte Carlo (MCMC) model for hypertension across individual, state, district, and cluster among reproductive aged (15-49 years) adults in India in 2016 and 2021.

| **Hypertension** | **Descriptives** | | | | **MCMC** | | | | | |
| --- | --- | --- | --- | --- | --- | --- | --- | --- | --- | --- |
| **2016 Women** | n | minimum | average | maximum | coef | Std Dev | ESS | 95% CI | Other MCMC | |
|  |  |  |  |  |  |  |  |  |  |  |
| **Constant** |  |  |  |  | -2.260583 | 0.0536677 | 2088 | (-2.37 - -2.15) | dbar | 399843.69 |
| **State** | 36 | 659 | 18349.4 | 95634 | 0.0911209 | 0.0250853 | 1565 | (0.05 - 0.15) | thetabar | 392868.11 |
| **District** | 640 | 230 | 1032.2 | 3327 | 0.0715166 | 0.0052046 | 1919 | (0.06 - 0.08) | effective no. of pars | 6975.58 |
| **Cluster** | 28351 | 1 | 23.3 | 57 | 0.1648903 | 0.0058627 | 1141 | (0.15 - 0.18) | B DIC | 406819.27 |
| **2016 Men** |  |  |  |  |  |  |  |  |  |  |
|  |  |  |  |  |  |  |  |  |  |  |
| **Constant** |  |  |  |  | -1.831344 | 0.0708327 | 1786 | (-1.97 - 1.70) | dbar | 73682.07 |
| **State** | 36 | 111 | 2672.8 | 12647 | 0.1551817 | 0.0464287 | 1963 | (0.09 - 0.27) | thetabar | 71640.24 |
| **District** | 640 | 31 | 150.3 | 432 | 0.0787028 | 0.0093915 | 955 | (0.06 - 0.10) | effective no. of pars | 2041.83 |
| **Cluster** | 9774 | 1 | 9.8 | 34 | 0.2090713 | 0.0168339 | 841 | (0.18 - 0.24) | B DIC | 75723.9 |
| **2021 Women** | |  |  |  |  |  |  |  |  |  |
|  |  |  |  |  |  |  |  |  |  |  |
| **Constant** |  |  |  |  | -2.229061 | 0.0547057 | 897 | (-2.34 - -2.12) | dbar | 411179 |
| **State** | 36 | 647 | 18105.8 | 85965 | 0.0969806 | 0.0276062 | 502 | (0.06 - 0.16) | thetabar | 401869.03 |
| **District** | 707 | 186 | 921.9 | 1615 | 0.0592978 | 0.00436 | 598 | (0.05 - 0.07) | effective no. of pars | 9309.97 |
| **Cluster** | 29828 | 1 | 21.9 | 48 | 0.2427318 | 0.0064158 | 637 | (0.23 - 0.26) | B DIC | 420488.97 |
| **2021 Men** |  |  |  |  |  |  |  |  |  |  |
|  |  |  |  |  |  |  |  |  |  |  |
| **Constant** |  |  |  |  | -1.680509 | 0.0738227 | 2053 | (-1.83 - 1.53) | dbar | 67648.17 |
| **State** | 36 | 85 | 2255.4 | 9926 | 0.1723008 | 0.0503549 | 2045 | (0.10 - 0.29) | thetabar | 65298.09 |
| **District** | 707 | 2 | 114.8 | 218 | 0.0737405 | 0.0097111 | 673 | (0.06 - 0.09) | effective no. of pars | 2350.08 |
| **Cluster** | 8910 | 1 | 9.1 | 25 | 0.2876996 | 0.0193291 | 804 | (0.25 - 0.33) | B DIC | 69998.25 |

**Table S2**: Markov Chain Monte Carlo (MCMC) model for diabetes across individual, state, district, and cluster among reproductive aged (15-49 years) adults in India in 2016 and 2021.

| **Diabetes** | **Descriptives** | | | | **MCMC** | | | | | |
| --- | --- | --- | --- | --- | --- | --- | --- | --- | --- | --- |
| **2016 Women** | n | minimum | average | maximum | coef | Std Dev | ESS | 95% CI | Other MCMC | |
|  |  |  |  |  |  |  |  |  |  |  |
| **Constant** |  |  |  |  | -4.710734 | 0.0904133 | 1826 | (-4.88 - -4.53) | dbar | 72434.03 |
| **State** | 36 | 657 | 18988.4 | 96228 | 0.2566 | 0.0745382 | 1721 | (0.15 - 0.43) | thetabar | 70478.02 |
| **District** | 640 | 390 | 1068.1 | 3324 | 0.1323443 | 0.0149092 | 1049 | (0.11 - 0.16) | effective no. of pars | 1956.01 |
| **Cluster** | 28456 | 1 | 24 | 57 | 0.2910346 | 0.0324733 | 270 | (0.22 - 0.35) | B DIC | 74390.04 |
| **2016 Men** |  |  |  |  |  |  |  |  |  |  |
|  |  |  |  |  |  |  |  |  |  |  |
| **Constant** |  |  |  |  | -4.396349 | 0.0943212 | 698 | (-4.58 - -4.22) | dbar | 13969.16 |
| **State** | 36 | 110 | 2774.9 | 12687 | 0.1808671 | 0.0597444 | 1743 | (0.09 - 0.32) | thetabar | 13390.38 |
| **District** | 640 | 31 | 156.1 | 432 | 0.0453468 | 0.0297022 | 9 | (0.00 - 0.10) | effective no. of pars | 578.78 |
| **Cluster** | 9859 | 1 | 10.1 | 31 | 0.4509725 | 0.1047211 | 155 | (0.25 - 0.66) | B DIC | 14547.94 |
| **2021 Women** | |  |  |  |  |  |  |  |  |  |
|  |  |  |  |  |  |  |  |  |  |  |
| **Constant** |  |  |  |  | -4.612589 | 0.114344 | 1949 | (-4.84 - -4.38) | dbar | 81635.02 |
| **State** | 36 | 630 | 19187.4 | 87384 | 0.4345117 | 0.126699 | 2234 | (0.25 - 0.76) | thetabar | 79895.33 |
| **District** | 707 | 181 | 977 | 1614 | 0.1476821 | 0.0150536 | 947 | (0.12 - 0.18) | effective no. of pars | 1739.69 |
| **Cluster** | 30113 | 1 | 22.9 | 49 | 0.2003324 | 0.0289221 | 222 | (0.15 - 0.26) | B DIC | 83374.71 |
| **2021 Men** |  |  |  |  |  |  |  |  |  |  |
|  |  |  |  |  |  |  |  |  |  |  |
| **Constant** |  |  |  |  | -4.312369 | 0.1226157 | 1122 | (-4.55 - -4.07) | dbar | 13261.51 |
| **State** | 36 | 85 | 2408.1 | 10171 | 0.3813137 | 0.1207055 | 2201 | (0.20 - 0.67) | thetabar | 12888.25 |
| **District** | 707 | 2 | 122.6 | 222 | 0.0765566 | 0.0370532 | 52 | (0.01 - 0.16) | effective no. of pars | 373.27 |
| **Cluster** | 9064 | 1 | 9.6 | 25 | 0.2330807 | 0.1093885 | 76 | (0.04 - 0.45) | B DIC | 13634.78 |

**Table S3**: Distribution of sample of hypertension and diabetes among reproductive aged (15-49 years) among states and union territories of India in 2016 and 2021

|  |  | **Hypertension** | | | | **Diabetes** | | | |
| --- | --- | --- | --- | --- | --- | --- | --- | --- | --- |
|  |  | **Female** | | **Male** | | **Female** | | **Male** | |
| **State** | Number of Districts | 2016 | 2021 | 2016 | 2021 | 2016 | 2021 | 2016 | 2021 |
| Andhra Pradesh | 26 | 9627 | 10171 | 1272 | 1281 | 9453 | 10452 | 1260 | 1290 |
| Arunachal Pradesh | 20 | 9045 | 18610 | 1129 | 2386 | 13436 | 19248 | 1772 | 2466 |
| Assam | 33 | 25708 | 28187 | 3393 | 3597 | 27395 | 33863 | 3675 | 4324 |
| Bihar | 38 | 45135 | 40478 | 5329 | 4134 | 45292 | 40888 | 5351 | 4188 |
| Chhattisgarh | 27 | 24779 | 27240 | 3446 | 3658 | 24803 | 27426 | 3444 | 3654 |
| Goa | 2 | 1688 | 742 | 760 | 89 | 1686 | 1950 | 760 | 256 |
| Gujarat | 33 | 21832 | 31083 | 5186 | 4537 | 22221 | 32509 | 5309 | 4747 |
| Haryana | 22 | 20929 | 20175 | 3261 | 2660 | 21355 | 20455 | 3319 | 2724 |
| Himachal Pradesh | 12 | 9249 | 10170 | 1976 | 1291 | 9684 | 10161 | 2106 | 1282 |
| Jharkhand | 24 | 28558 | 25387 | 3699 | 2930 | 28489 | 25178 | 3688 | 2897 |
| Karnataka | 30 | 23935 | 24814 | 3275 | 3197 | 25524 | 29078 | 3553 | 3725 |
| Kerala | 14 | 10496 | 10201 | 1731 | 1142 | 10821 | 10448 | 1816 | 1208 |
| Madhya Pradesh | 51 | 61466 | 45282 | 9215 | 5913 | 61829 | 45015 | 9267 | 5855 |
| Maharashtra | 36 | 27944 | 30486 | 4089 | 4310 | 28643 | 32267 | 4199 | 4632 |
| Manipur | 9 | 13172 | 7511 | 1674 | 978 | 13531 | 7898 | 1723 | 1035 |
| Meghalaya | 11 | 8739 | 11506 | 1068 | 1453 | 8939 | 12720 | 1082 | 1655 |
| Mizoram | 8 | 12123 | 7119 | 1586 | 992 | 12146 | 7111 | 1588 | 988 |
| Nagaland | 11 | 9713 | 6425 | 1218 | 864 | 10382 | 9466 | 1299 | 1267 |
| Odisha | 30 | 27348 | 26932 | 3275 | 3286 | 33087 | 27172 | 4036 | 3327 |
| Punjab | 22 | 19172 | 19726 | 2972 | 2620 | 19174 | 19795 | 2971 | 2671 |
| Rajasthan | 33 | 41536 | 40807 | 5799 | 5372 | 41507 | 41715 | 5797 | 5567 |
| Sikkim | 4 | 4849 | 2817 | 729 | 378 | 5262 | 3052 | 796 | 395 |
| Tamil Nadu | 32 | 27015 | 24881 | 4485 | 2868 | 28530 | 24735 | 4696 | 2827 |
| Telangana | 31 | 6980 | 23851 | 964 | 2864 | 6983 | 25728 | 964 | 3137 |
| Tripura | 8 | 4681 | 6931 | 792 | 852 | 4647 | 7137 | 784 | 875 |
| Uttar Pradesh | 75 | 95658 | 85965 | 12647 | 9926 | 96255 | 87384 | 12687 | 10171 |
| Uttarakhand | 13 | 16864 | 12295 | 1913 | 1273 | 16946 | 12470 | 1914 | 1288 |
| West Bengal | 20 | 16552 | 17539 | 2187 | 2210 | 17067 | 20646 | 2307 | 2559 |
| **Union Territories** |  |  |  |  |  |  |  |  |  |
| Andaman & Nicobar Islands | 3 | 1802 | 2088 | 270 | 266 | 2794 | 2340 | 408 | 327 |
| Chandigarh | 1 | 659 | 647 | 111 | 85 | 657 | 630 | 110 | 85 |
| Dadra & Nagar Haveli and Daman & Diu | 3 | 2086 | 2145 | 580 | 285 | 2069 | 2615 | 578 | 372 |
| Jammu & Kashmir | 20 | 20679 | 13227 | 4764 | 1404 | 21571 | 22282 | 5040 | 2745 |
| Ladakh | 2 | 1729 | 1511 | 345 | 173 | 1832 | 2302 | 374 | 274 |
| Lakshadweep | 1 | 945 | 1213 | 117 | 121 | 1063 | 1202 | 149 | 121 |
| NCT of Delhi | 11 | 4103 | 10053 | 400 | 1355 | 4519 | 9814 | 465 | 1318 |
| Puducherry | 4 | 3784 | 3594 | 565 | 443 | 3990 | 3596 | 609 | 440 |

**Figure S1**: District-level prevalence of hypertension among reproductive aged women (15-49 years) in India, 2016.


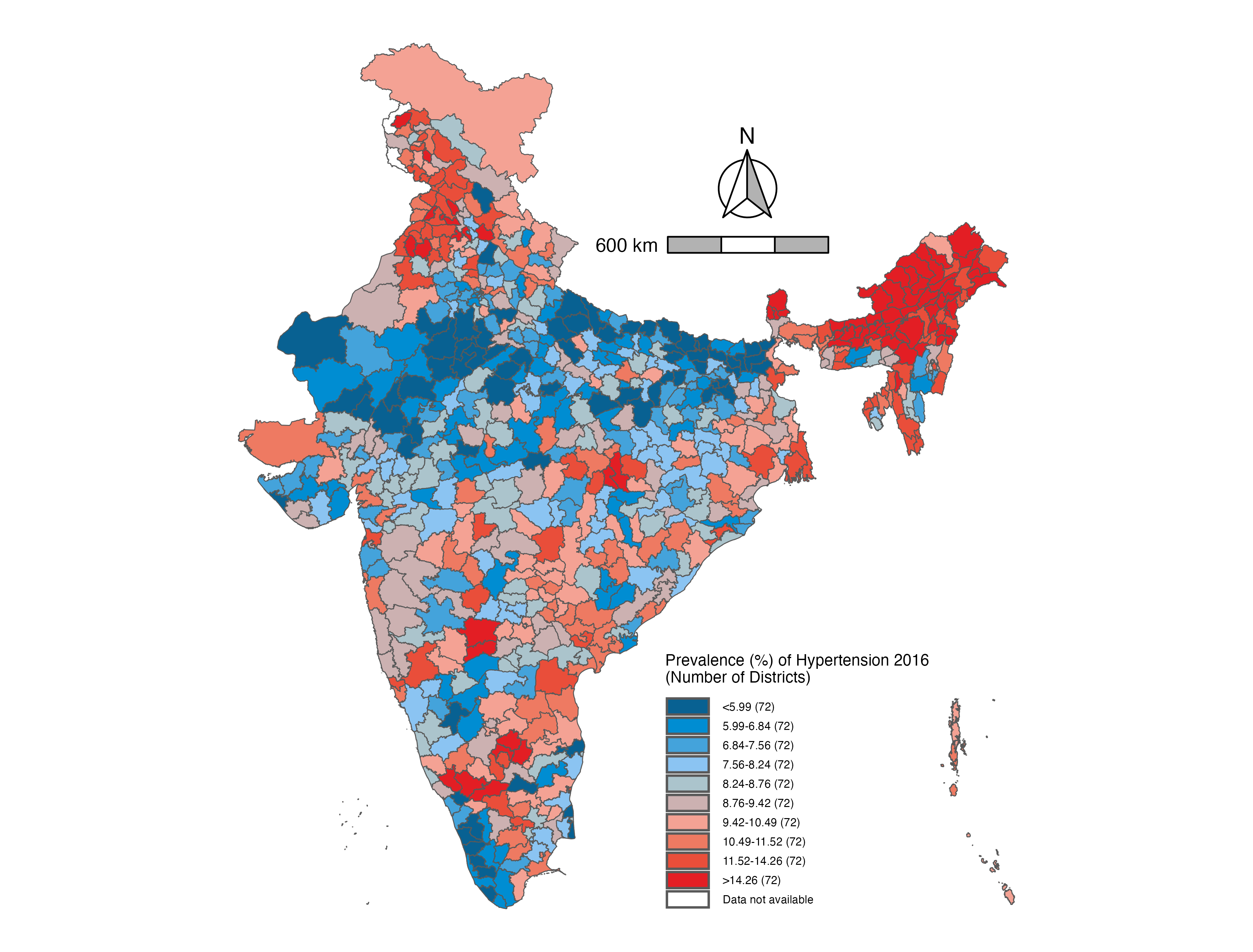


**Figure S2**: District-level prevalence of hypertension among reproductive aged men (15-49 years) in India, 2016.


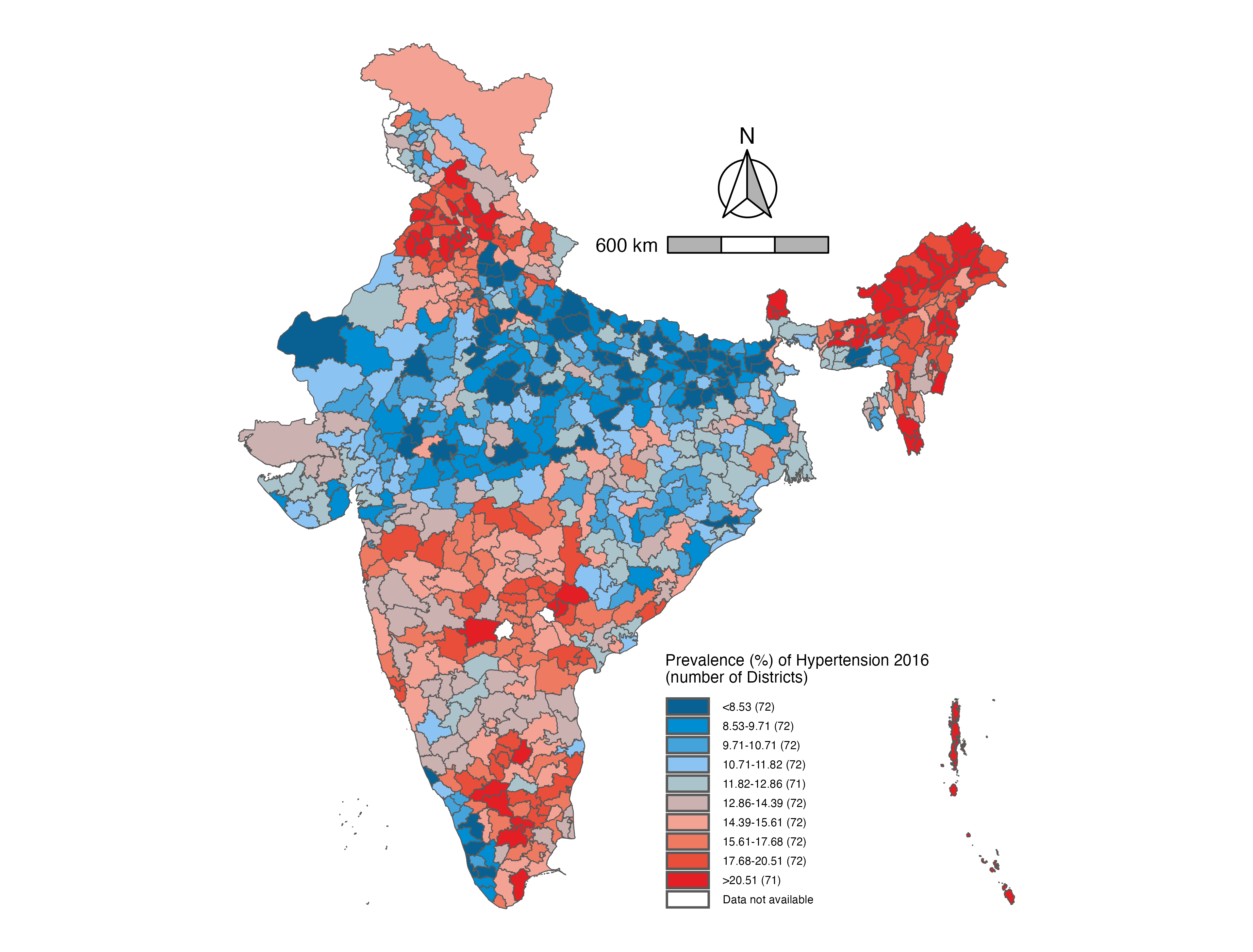


**Figure S3**: District-level prevalence of diabetes among reproductive aged women (15-49 years) in India, 2016.


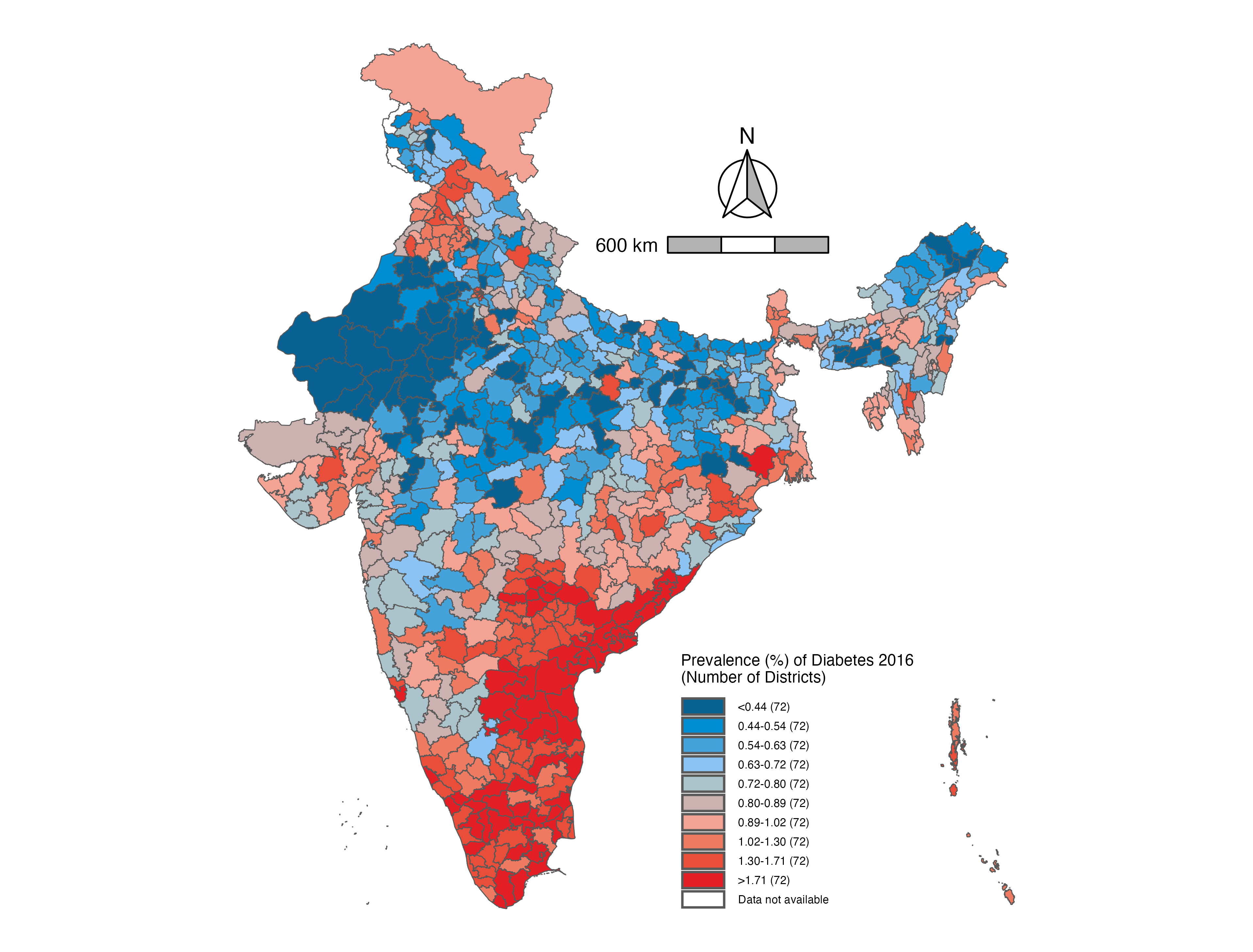


**Figure S4**: District-level prevalence of diabetes among reproductive aged men (15-49 years) in India, 2016.


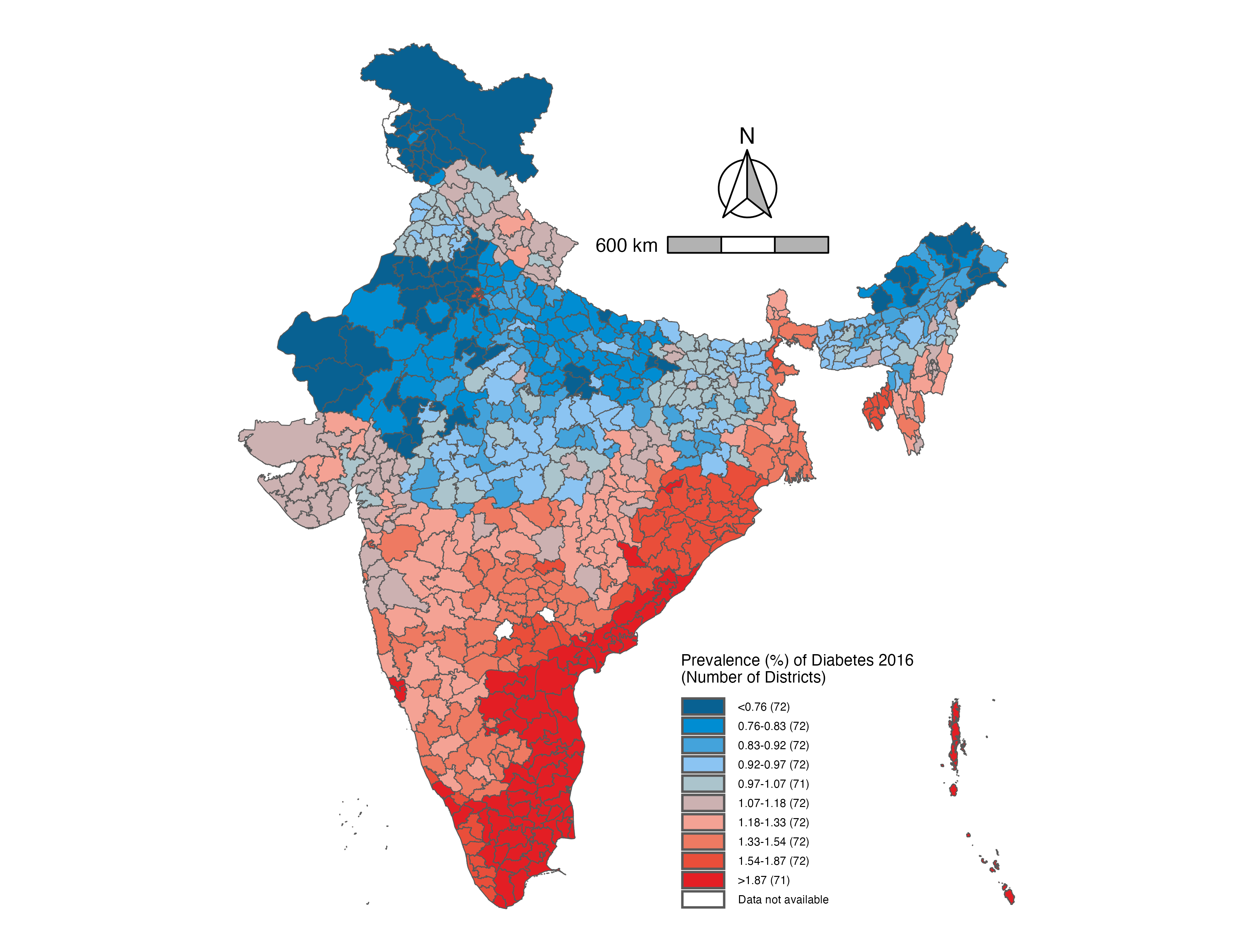


**Figure S5**: District level association between prevalence of hypertension and diabetes among reproductive aged women (15-49) in 2021.


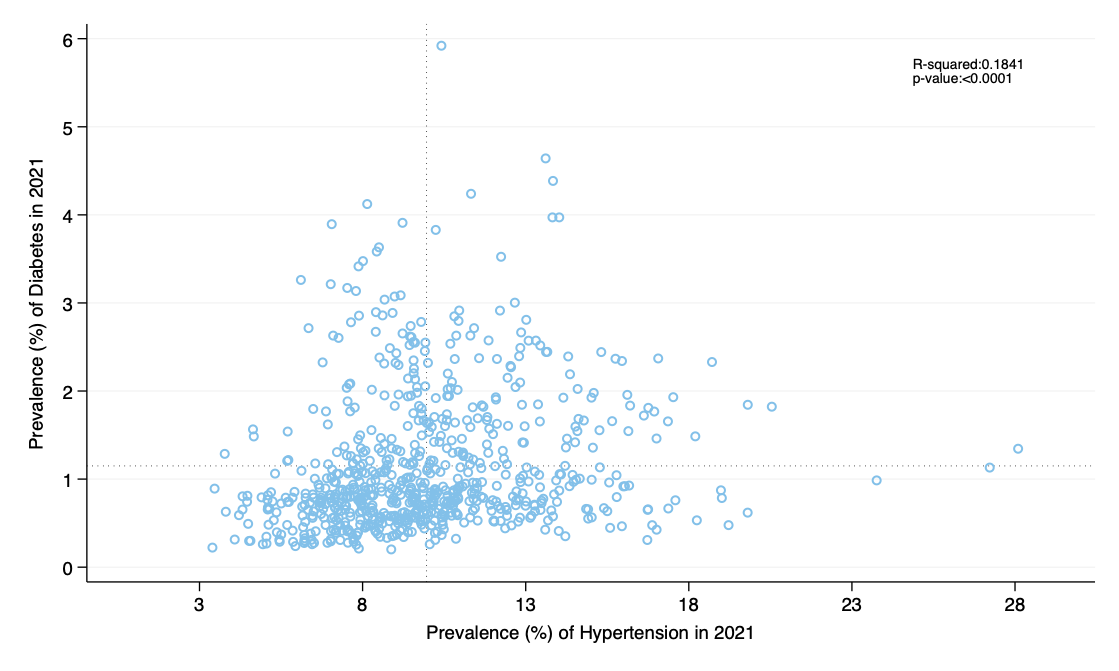


**Figure S6**: District level association between prevalence of hypertension and diabetes among reproductive aged women (15-49) in 2016.


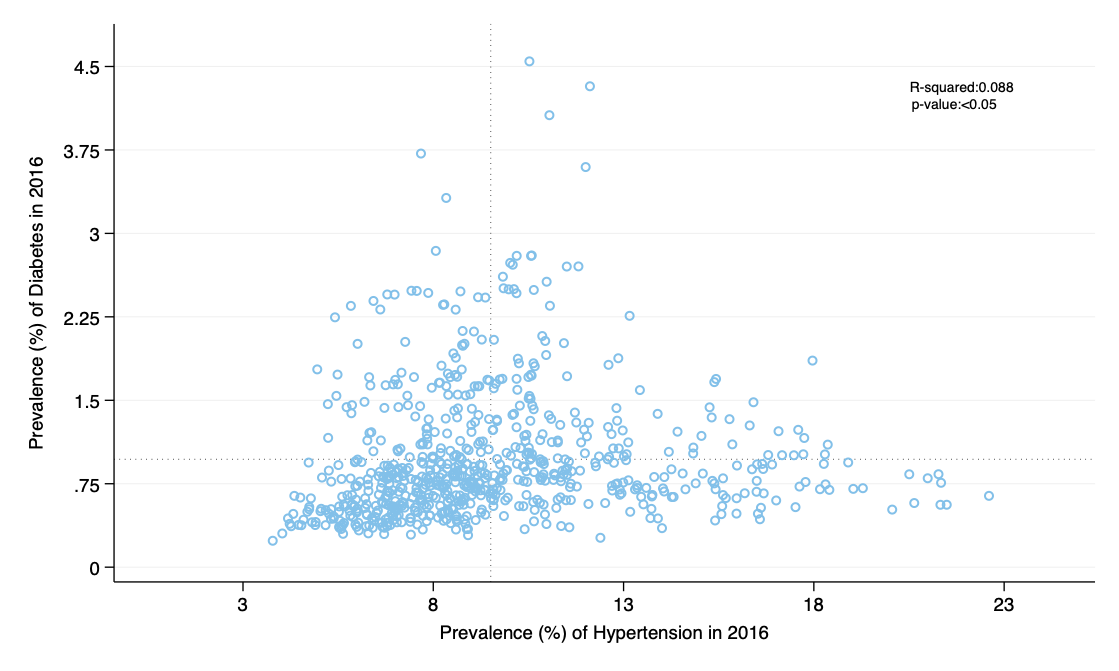


**Figure S7**: District level association between prevalence of hypertension and diabetes among reproductive aged men (15-49) in 2021.


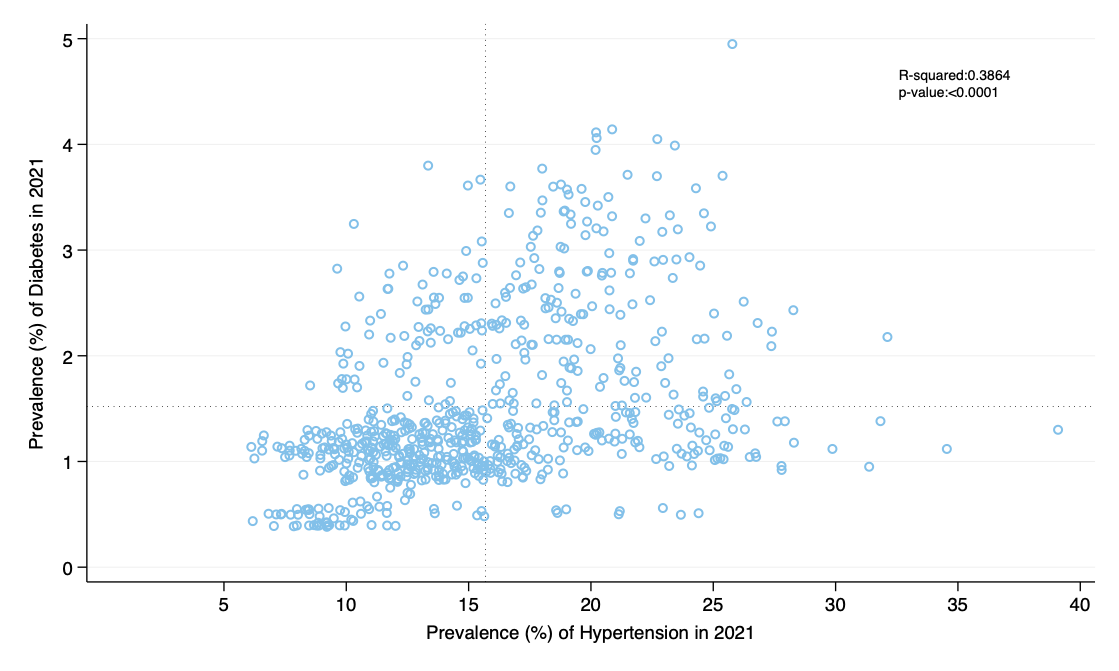


**Figure S8**: District level association between prevalence of hypertension and diabetes among reproductive aged men (15-49) in 2016.


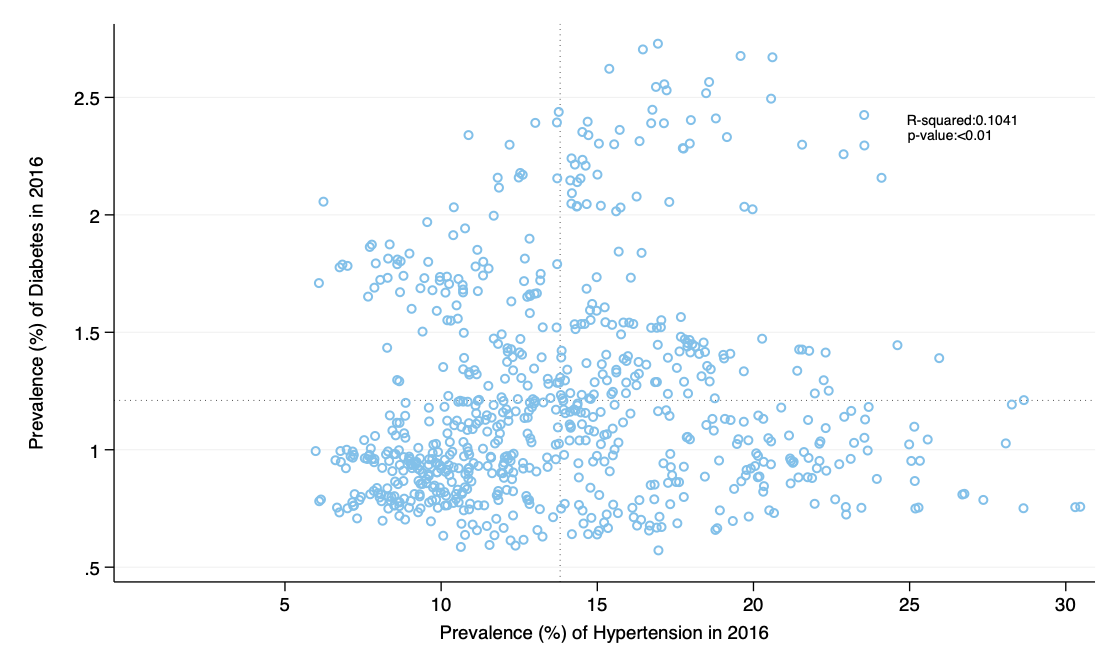

Supplement: online supplemental file 1 [file bmjph-3-2-s001.docx]
